# The FEL in the SXL project at MAX IV

**DOI:** 10.1107/S1600577521003465

**Published:** 2021-04-23

**Authors:** Weilun Qin, Francesca Curbis, Joel Andersson, Vitaliy Goryashko, Lennart Isaksson, Billy Kyle, Filip Lindau, Erik Mansten, Mihai Pop, Peter Salén, Hamed Tarawneh, Pedro F. Tavares, Sara Thorin, Alexey Vorozhtsov, Sverker Werin

**Affiliations:** a Department of Physics, Lund University, PO Box 118, SE-22100 Lund, Sweden; b MAX IV Laboratory, Lund University, PO Box 118, SE-22100 Lund, Sweden; c FREIA Laboratory, Department of Physics and Astronomy, Uppsala University, PO Box 516, SE-75120 Uppsala, Sweden

**Keywords:** free-electron laser, soft X-ray, MAX IV, two-color/two-pulses

## Abstract

In this paper the design of the free-electron laser (FEL) in the SXL (Soft X-ray Laser) project at the MAX IV Laboratory is presented. The target performance parameters originate in a science case put forward by Swedish users and the SXL FEL is foreseen to be driven by the existing MAX IV 3 GeV linac. The SXL project is planned to be realized in different stages and in this paper the focus is on Phase 1, where the basic operation mode for the FEL will be SASE (self-amplified spontaneous emission), with an emphasis on short pulses. Simulation results for two linac bunches (high and low charge) with different pulse duration are illustrated, as well as the performance for two-color/two-pulses mode and power enhancement through tapering. Besides standard SASE and optical klystron configurations, the FEL setup is also tailored to allow for advanced seeding schemes operations. Finally possible upgrades that will be implemented in a second phase of the project are discussed.

## Abstract 2

The design of a soft X-ray free-electron laser for the SXL project at the MAX IV Laboratory is presented.

## 1. Introduction   

In 2016 a workshop was held in Stockholm gathering both Swedish and foreign scientists to discuss the case of a Swedish free-electron laser (FEL) project (Nilsson, 2016 ▸), called SXL (Soft X-ray Laser). The approach was to explore the wavelength window between the LCLS (Emma *et al.*, 2010 ▸) and the European VUV facilities FERMI (Allaria *et al.*, 2012 ▸) and FLASH (Ayvazyan *et al.*, 2006 ▸). The outcome of the workshop was a broad interest for a medium-size FEL targeting the 1–5 nm (∼0.25–1.2 keV) range and built immediately on the existing 3 GeV MAX IV linear accelerator, especially regarding the accelerator already in operation and the emerging experiences with its Short-Pulse Facility (SPF) and its first beamline, the FemtoMAX (Enquist *et al.*, 2018 ▸).

In the meantime the development of the Athos beamline at SwissFEL (Reiche *et al.*, 2019 ▸) reflects similar demand from a portion of the FEL user community. There is a partial overlap in photon energy with the SASE3 beamline at European XFEL (Saldin *et al.*, 2009 ▸; Liu *et al.*, 2019 ▸), and also FLASH (Beye & Klumpp, 2020 ▸) and FERMI (Fabris *et al.*, 2016 ▸) have upgrade plans that are targeting the same range while SXFEL (Zhao *et al.*, 2017 ▸) in Shanghai is slightly shifted towards longer wavelengths.

In 2018 a design study was funded by the Knut and Alice Wallenberg (KAW) foundation together with several Swedish universities and the MAX IV Laboratory.

In the continuing work with the science case a set of representative experiments was selected, in order to define the requirements for the FEL design, grouped in: atomic, molecular and optical (AMO) physics, condensed matter, chemistry and imaging in life science. The common leitmotiv of the various experiments in the science case is the availability of a wide range of pump sources already embedded in the SXL design. The AMO experiments will explore processes from charge migration to charge transfer using ultra-short pulses and two pulses with different colors in order to implement pump–probe schemes. For chemistry the main focus is in understanding the dynamics of heterogeneous catalysis and probing transition states in surface reactions, where variable polarization is desired. In condensed matter the goal is to create new phases in quantum materials (like strontium titanate) with THz radiation and probe the emergent order with the FEL beam. Also experiments on coherence control in the attosecond frontier are foreseen, and they will require sub-femtosecond pulses, in addition to full control of the FEL polarization. Pumping with an HHG (high harmonic generation) source will also be beneficial. In life science the combination of THz or other pump lasers with the FEL radiation will allow to probe conformational changes in solutions by scattering experiments. Relatively long (order of 20 fs) pulses with two colors will be desired together with a split-and-delay line for dynamics between 100 fs and 10 ms.

The injector system at the MAX IV Laboratory has been conceived from the beginning to be able to also drive an FEL, besides injecting into the two storage rings and the short-pulse facility (MAX-lab, 2010 ▸). The FEL design is based on two distinct modes of operation for the linear accelerator: high-charge/medium-compression and low-charge/strong-compression. The SXL will come in two phases, where the first phase will satisfy the bulk of the user requirements in SASE mode with full polarization control, two-pulse/two-color operation, enhanced power through tapering and short pulses below 10 fs. In a second phase more advanced concepts are envisaged: ultra-short pulses (few tens of attoseconds), coherence enhancement and seeding.

In this paper we present the design of a soft X-ray FEL. After a description of the linear accelerator, we will focus on the FEL design, followed by detailed performance for different FEL operation modes. A short description of the optical beamline is then presented. Finally, outlook and upgrades opportunities are discussed.

## 2. Injector and linac   

The accelerator system today at MAX IV Laboratory comprises two guns (a thermionic RF gun and a photo-cathode gun), a 3 GeV S-band linear accelerator with two bunch compressors placed at, respectively, about 270 MeV (depending on the compression) and full energy. For storage rings injections, the electron beam generated in the thermionic gun can be extracted at 1.5 GeV and 3 GeV. The photo-cathode gun, with emittance compensation, is used for SPF operation (Enquist *et al.*, 2018 ▸) and automated switching is already in place to allow top-up ring injections.

**Figure 1:**
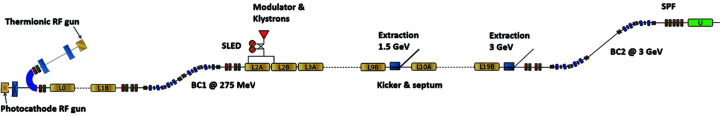
General layout of the linear accelerator that will drive the SXL.

In the SPF hall there are currently two beamlines, the FemtoMAX (Enquist *et al.*, 2018 ▸) and a diagnostics beamline (Tavares *et al.*, 2019 ▸). The SXL will be built in one of the empty available beamline slots and extended beyond the current SPF hall to house the optical beamline and endstations. The layout of the existing MAX IV linac is presented in Fig. 1 ▸.

The FEL operation modes planned to address the science case will be fed by two basic accelerator modes (called 1A and 1B) that will be available in the first phase of the project. The two modes differ mainly on the charge and compression and consequently on the bunch duration at the undulator entrance. The main bunch parameters for the two modes are summarized in Table 1 ▸. Variations to these main modes are foreseen to match more detailed requirements for specific experiments.

The two modes, at 100 pC and 10 pC charge, respectively, have been chosen as they fulfill the relevant user cases. While preliminary studies have been made on 200 pC, increasing the pulse charge also gives new challenges in electron beam quality and handling. As there is no immediate need for such a mode in the user case, we have chosen not to study higher charges at this stage.

The injector system is already today capable of delivering an electron pulse able to drive an FEL with basic performance. With minor improvements the system will become an excellent FEL driver with unique capabilities, such as naturally short electron pulses, passive linearization in the bunch compressors and a low jitter. Improvements already under way are an upgrade of the photo-cathode gun to a 100 Hz system and the completion of the diagnostics beamline for full longitudinal phase space reconstruction (Tavares *et al.*, 2019 ▸).

The improvements to the accelerator system will mainly be performed in two areas: optics and diagnostics. To allow a better control during the compression to ultra short pulses, the CSR (coherent synchrotron radiation) effects have been minimized through better optimization of the magnet lattice which provides improved control of the emittance growth. In order to better characterize the beam at an early stage, it is planned for a longitudinal diagnostics station before the first bunch compressor (BC1). In addition, a laser heater (Huang *et al.*, 2004 ▸, 2010 ▸) option to mitigate microbunching instabilities was designed and integrated into the accelerator.

Two double-achromat compressor structures allow to compress the electron pulses emitted by the photo-cathode gun from a few picoseconds to tens of femtoseconds or even less. The bunch compressors are able to passively linearize the longitudinal phase space, namely to avoid the necessity of a higher-order harmonic cavity (Thorin *et al.*, 2010 ▸). Since the compressors have a positive *R*_56_, the electron beam needs to be accelerated on the negative slope of the RF sine wave and thus the beam displays a positive energy chirp, in contrast to the chirp needed in a regular chicane bunch compressor. This type of chirp is more difficult to remove, as the usual methods of de-chirping will actually increase the chirp (Bane & Stupakov, 2012 ▸; Emma *et al.*, 2014 ▸; Zhang *et al.*, 2015 ▸). An additional difference from many other FEL drivers is that the last compression stage is done at full energy. At the time of writing, we are studying ways of coping/removing the chirp. A promising path that will be studied further is overcompression of the pulse in the last bunch compressor, resulting in a reversed chirp, which then can be removed by a traditional dechirper. Initial results indicate a 40 fs flat pulse with just below 1 kA current. Other options include compressing mainly in the first bunch compressor or using a dielectic dechirper.

As the double-achromat bunch compressors require operating on the reverse RF slope, as compared with chicane compressors, the RF modulator voltage jitter sensitivity is reduced as the resulting phase and amplitude jitter counteract each other.

**Figure 2:**
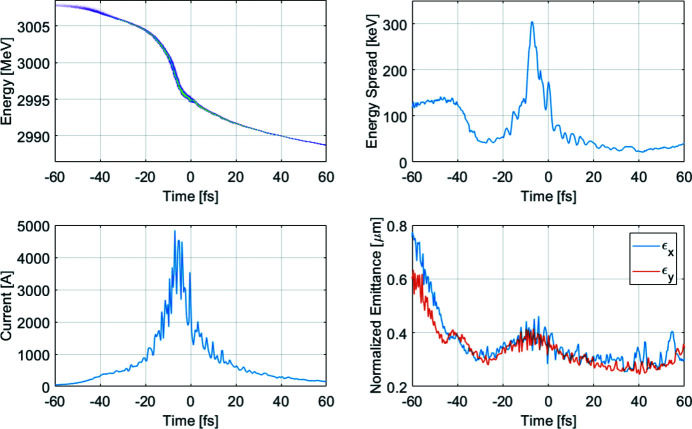
Longitudinal phase space, energy spread, current and emittance at the entrance of the undulator for the long pulse case (1A).

Start-to-end particle tracking has been performed to investigate the electron properties. The electron beams are tracked through the accelerator using *ASTRA* (Flöttmann *et al.*, 2011 ▸) for the injector part up to about 100 MeV, and then using *ELEGANT* (Borland, 2000 ▸) for the main linac and bunch compressors until the entrance of the undulator. The slice parameters and longitudinal phase space at the entrance of the undulator for the high-charge medium-compression/long pulse mode (1A) are shown in Fig. 2 ▸. The bunch duration is about 11 fs (FWHM), the energy chirp 0.4 MeV fs^−1^ and the slice RMS normalized emittance is below 0.4 mm mrad. The peak current is about 4500 A. This beam has been obtained with 100 pC charge from the gun and compression factors 11 and 6 (from RMS values) in the first and the second bunch compressors, respectively. The high charge bunch aims for producing FEL pulses with large number of photons and moderate bunch duration. The bunch displays an intrinsic energy chirp which has a small impact on the FEL performance (see next section[Sec sec3]).

**Figure 3:**
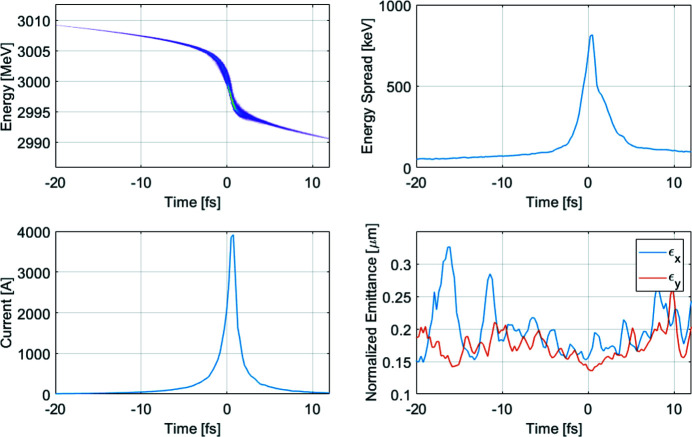
Longitudinal phase space, energy spread, current and emittance at the entrance of the undulator for the short pulse case (1B). (Emittance variations at the edges of the beam are due only to poor statistics.)

The slice parameters and longitudinal phase space at the entrance of the undulator for the short pulse mode (1B) are shown in Fig. 3 ▸. The beam is obtained with 10 pC charge at the gun, and compressed by a factor of 5 and 38 in the two bunch compressors, respectively. The peak current is about 3900 A and the pulse duration is about 1.3 fs (FWHM), the energy chirp is 5 MeV fs^−1^ and the slice RMS emittance is about 0.2 mm mrad.

## 3. FEL design   

The science case for the SXL requires a full control of the polarization and ultra-short pulses. In a second phase the FEL design needs the flexibility to introduce advanced concepts to address coherence enhancement and seeding.

The case that is shown is ambitious in regard of electron beam performance, but redundancy to handle a less ideal situation is in most cases available through a larger number of undulator modules available, than utilized in the simulations.

The FEL design thus relies on 2 m-long APPLE-X type undulators separated by drift sections. The undulator design is very compact (Tarawneh *et al.*, 2019 ▸) and will allow tunability between 5 and 1 nm (about 250 and 1240 eV), with the undulator *K* value varying from 1.2 to 3.9, using fixed 3 GeV electron energy. A summary of the undulator parameters is given in Table 2 ▸. We envisage 20 undulators, which correspond to a total active magnetic length of 40 m, with a 5 m-long break after 10 modules in order to accommodate a big magnetic chicane which can serve multiple purposes.

**Figure 4:**
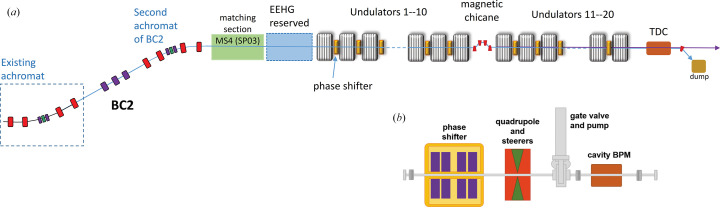
(*a*) Layout of the SXL undulator line, and (*b*) layout of the undulator intrasection.

In Phase 1, the chicane will be used to adjust the delays between two FEL pulses in two-color/two-pulse FEL generation. In Phase 2, the chicane could also be used to bypass the electron beams for the implementation of a soft X-ray monochromator for self-seeding. A sketch of the FEL section layout is shown in Fig. 4(*a*) ▸.

The undulator line will start next to the current FemtoMAX beamline in the SPF hall. The current building will be further extended to house the undulators, the beamline and the experimental stations. Prior to the undulator section, *i.e.* at the end of the second bunch compressor, a space is reserved for beam manipulation before the radiating undulators. Besides a matching section, this part is prepared to house modulators and chicanes to allow the implementation of echo-enabled harmonic generation (EEHG) seeding (Pop & Werin, 2019 ▸). The FEL will be followed by a transverse deflecting cavity system for post-FEL longitudinal diagnostics, and a beam dump.

The undulator intrasection, Fig. 4(*b*) ▸, is planned to be 0.76 m, which can, with negligible impact on the FEL performance, be increased up to 1 m to allow adjustments of optics and diagnostics in the intrasection. The layout will include a quadrupole, to provide a FODO lattice, with integrated steerers, a small magnetic chicane for both phase shift and pulse delay, a cavity BPM and a gate valve with a pumping port. The compact chicanes, providing delays up to 6 fs, are also used to shift the electron beam for advanced FEL schemes such as HB-SASE and attosecond pulse trains (Reiche *et al.*, 2019 ▸; Mak *et al.*, 2019 ▸) and could also be used to displace the electron beam horizontally.

## 4. Long pulses   

In Phase 1, the SXL operations will be based on a SASE configuration, a mode that will satisfy the bulk of the proposed user activities. One of the main FEL operation modes is long pulse mode, utilizing the medium compressed electron beam (1A) in Fig. 2 ▸. The baseline FEL performance for the start-to-end long electron pulse has been investigated with the FEL simulation code *GENESIS* (Reiche, 1999 ▸).

**Figure 5:**
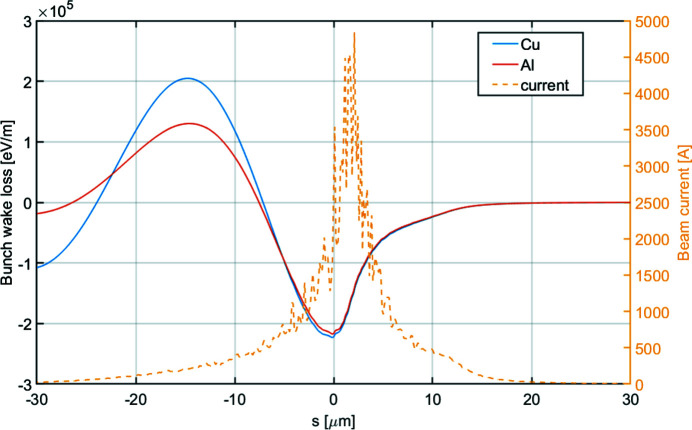
Resistive wall wakefield-induced energy loss of the SXL undulator for the long pulse.

As the electron beam travels through the long undulator vacuum pipe, the longitudinal wakefields will induce an additional energy loss and energy chirp to the electron beam. The SXL undulator pipe is round with an inner diameter of 5 mm. The corresponding resistive wall (bunch) wakefield induced energy loss for Al and Cu are shown in Fig. 5 ▸. The average wake loss experienced by the beam is about 120 keV m^−1^. Particularly for the SXL beam, the wake-induced energy chirp takes the same sign as the intrinsic chirp and thus further increases the chirp. The energy loss from the wakefields causes the electron beam energy to deviate from the resonant energy, which could be compensated by a linear taper starting from the beginning of the undulator. Compared with the intrinsic energy chirp, the wakefield-induced energy chirp is relatively small and thus no significant increase on the FEL bandwidth is expected due to wakefields.

**Figure 6:**
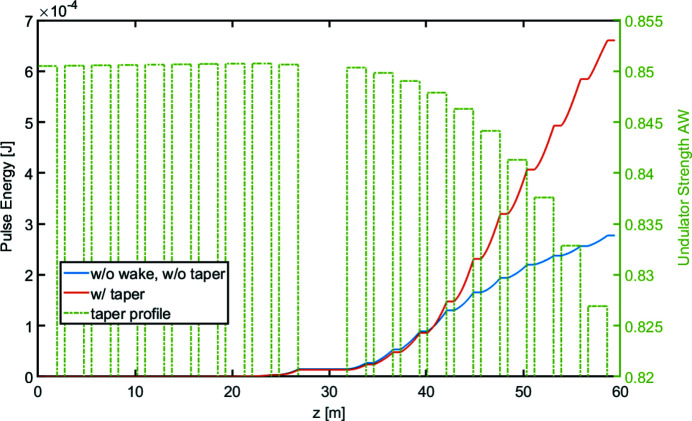
Undulator taper profile and pulse energy evolution for the long pulse at 1 nm.

The total active length of 30 m is sufficient to reach saturation for the shortest designed wavelength, 1 nm, leaving about 10 m of undulator available for post-saturation tapering to further enhance the FEL pulse energy. In our simulations, a quartic (fourth-order) taper profile, as shown in Fig. 6 ▸, has been optimized to maximize the output pulse energy as well as minimize the ‘effective emittance’ of the light.^1^ The undulator tapering increases the pulse energy to about 660 µJ, while the bandwidth remains about the same.

**Figure 7:**
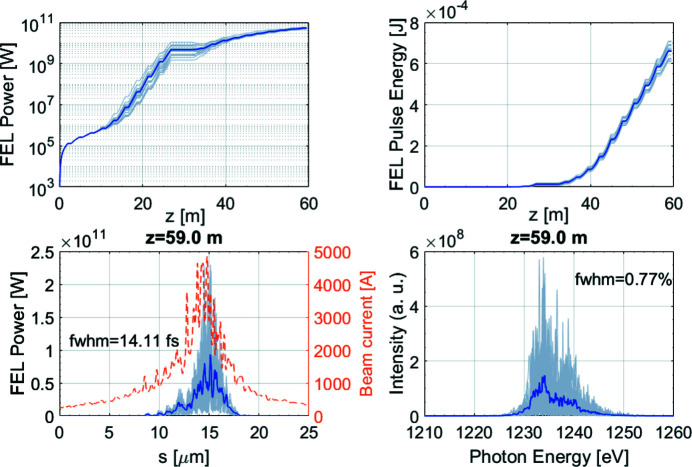
*GENESIS* simulated FEL performance for the start-to-end long pulse (1A) at 1 nm in SASE configuration with tapering. Each gray line corresponds to one-shot simulation and the blue line corresponds to the average of all the 20 shots.

Taking the undulator wakefields and tapering into account, multi-shot FEL performance at the shortest designed wavelength, 1 nm, is shown in Fig. 7 ▸. The produced FEL pulses are about 14 fs long in FWHM and have tens of GW power. Due to the residual beam energy chirp, the FEL bandwidth is broadened to about 0.8%.

Table 3 ▸ summarizes the FEL performance at 1 and 5 nm obtained using the long pulse (1A) with high charge (100 pC). While most of the quantities have been obtained through *GENESIS* simulations, for the last three (namely source size, source divergence and source location) the following method has been applied: the radiation field is dumped at the undulator exit and propagated for several tens of meters. A linear fit is used to determine the divergence and with back-propagation the waist size and waist location are retrieved. This explains the negative sign of the source location, which means that the virtual waist location is before the end of the undulator.

## 5. Short pulses   

Obtaining ultra-short FEL pulses is crucial for a subset of the experiments in the SXL science case, namely the time-resolved applications aiming at ultra-fast time scales. At the moment the requirements are on pulses of a couple of femtoseconds, but we believe the demand will grow for even shorter pulses, below 1 fs.

We have investigated a couple of methods to produce ultra-short pulses for the SXL. The baseline accelerator design allows to lower the bunch charge so that the injector can produce a much shorter initial electron bunch, which opens up for higher compression in the linac. The electron beam produced in such a way is shown as the short pulse case (1B) in Fig. 3 ▸.

**Figure 8:**
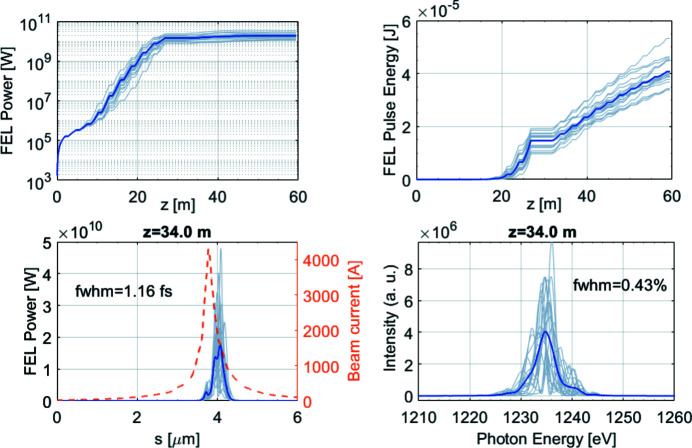
*GENESIS* simulated FEL performance for the start-to-end short pulse (1B) at 1 nm. Each gray line corresponds to one-shot simulation and the blue line corresponds to the average over 20 shots.

*GENESIS* simulations have been performed to investigate the performance of the short electron bunch. Figure 8 ▸ shows multiple shots SASE simulation results at 1 nm. The FEL pulses saturate after 11 modules, reaching about 17 µJ pulse energy. Thanks to the narrow current peak, the produced FEL pulses have a FWHM duration of 1.2 fs. Besides, the FEL pulse profile shows single-spike properties, indicating good longitudinal coherence. For longer radiation wavelength, the slippage effect becomes much more significant and the FEL pulse slips out of the electron bunch. To maintain a reasonable interaction length between the FEL pulse and the electron beam, the compression factor can optionally be decreased to generate a slightly longer electron bunch for 5 nm radiation. This comes at the expense of lower peak current, which reduces the efficiency. In this case the FEL pulse energy reaches about 40 µJ and the FWHM pulse duration is 1.7 fs. The saturation is reached after seven undulators (results not shown).

While the compression for the short pulse case (1B) is strong and potentially sensitive to jitter, we have also looked into other methods, such as using a scraper/slotted foil to select a short region of the bunch in the bunch compressors. This could be implemented in the second bunch compressor where the beam is transversely dispersed and a transverse collimation can be converted to longitudinal selection. So far these are back-up options and not pursued at the moment.

## 6. Two-color/two-pulses   

A strong user requirement is the possibility to have two pulses with the same or different wavelength with adjustable delay, the so-called ‘two-color/two-pulses’ mode. The users’ wishes regarding the delay are diverse, spanning from milliseconds down to femtoseconds, with the chance of overlapping the pulses and ‘cross them over’.

**Figure 9:**
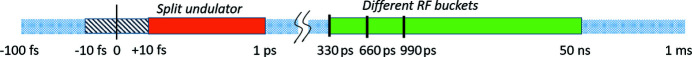
Two-color/two-pulses covered time intervals. In orange the split undulator which will cover from +10 fs to 1 ps, and in green the different RF buckets range.

This FEL mode needs to be implemented in different ways in different ranges and with different performances: split undulators (Lutman *et al.*, 2013 ▸), two electron pulses in the same RF bucket (Björklund Svensson *et al.*, 2019 ▸), two electron pulses in adjacent RF buckets (Decker *et al.*, 2015 ▸) and using a slotted-foil (Emma *et al.*, 2004 ▸). In Fig. 9 ▸ the possible time intervals that can be covered in SXL by two selected techniques are shown.

For long time separations (greater than a few picoseconds) the SXL will be operated with two electron pulses in adjacent RF buckets by illuminating the cathode with two laser pulses. The time interval comes in multiples of the RF frequency at *n* × 333 ps up towards a maximum delay of 50 ns. The 333 ps steps are from a user perspective adequate. The maximum delay is mainly limited by the filling time of the RF gun structure operated with SLED cavities (Kumbaro, 2015 ▸) which leave a flat-field region limited to 50 ns. The energy of the pulses will be defined by the difference in RF voltage and correlated to the delay. Tunability of the electron pulses will be limited (Björklund Svensson *et al.*, 2017 ▸) while from the FEL point of view these pulses can be regarded as more or less independent, and with similar performance.

For shorter time separations another scheme is the so-called ‘split undulator’ where the first portion of the undulators is tuned at one wavelength and the second to the same or another wavelength. By complementing the system with a chicane between the two undulator sections, the delay can be tuned from +10 fs to +1 ps. The complete overlap of the pulses and the cross-over (*i.e.* switching the order of the pulses) could be implemented at the beamline. Despite a rather simple concept, where the same electron bunch will produce the two photon pulses, the first pulse development has to be stopped well before saturation, otherwise the electron beam properties will be spoiled at the expense of the second color generation. This will couple the generation of the two pulses to each other, limiting the wavelength tuning range as the second undulator will use an electron bunch with increased energy spread making it difficult to reach shorter wavelengths. As the magnitude of the delay between the two pulses is coupled to the chicane strength, different time separations will erase the microbunches differently. Thus the FEL pulse energies will be coupled to time and wavelength separation.

**Figure 10:**
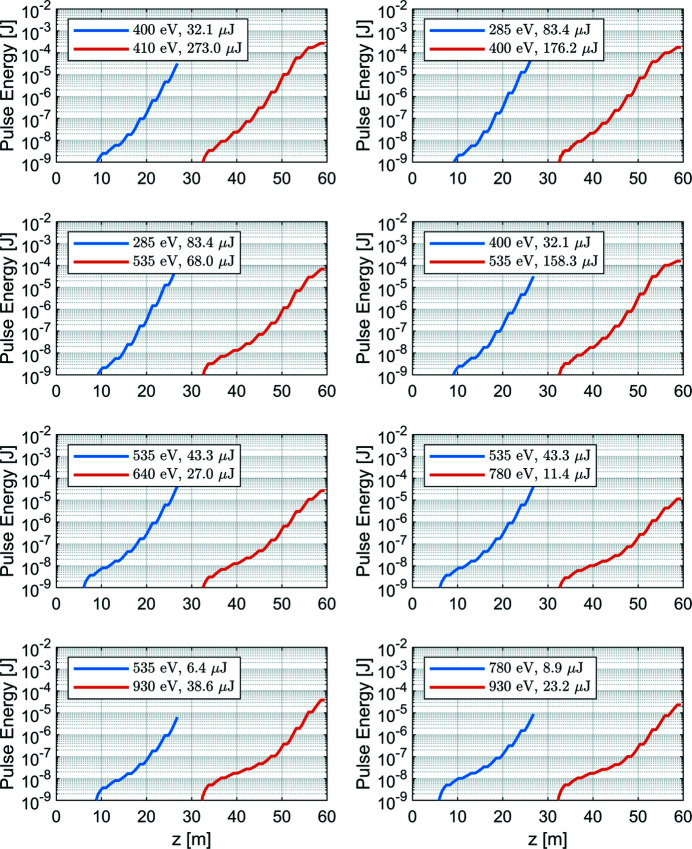
*GENESIS* simulated FEL performance for the two-color/two-pulses mode using the split undulator scheme. For the case with 400 eV and 410 eV, the chicane delay is set to be 200 fs; for other cases, the delays are set to 100 fs.

Simulations for the split undulator case have been performed using the long pulse (1A) for several combinations of delays and wavelength separations. The wavelength separations cover a wide range of interest from the potential experiments, including small separation down to a few eV and large separation targeting X-ray absorption edges of different atoms, up to a few hundred eV. Results for the two-color/two-pulse combinations are given in Fig. 10 ▸. The delays of the chicane are set to be large enough to erase the microbunching from the first stage. For the case with 400 eV and 410 eV, 200 fs delay is used. For the other cases with large wavelength separation, the delays are set to be 100 fs. Although there are 10 undulator modules for the first section, not all the 10 modules are used for the first color. The number of modules in the first section are selected individually for each combination to maintain enough electron beam quality for the lasing of the second color, but also generate sufficient pulse energy for the first color. The output pulse energies for the two pulses are comparable and are a few to a few hundred µJ, as shown in Fig. 10 ▸. Further tunability is still available by using different numbers of modules for the first or second color. For most of the two color combinations there is a safety margin to reach to desired pulse energy if the electron beam parameters are not at the design values and this is possible because, as already mentioned, not all 20 undulators are used. An exception (to this redundancy) is for the combination of very short wavelengths where 8 + 10 undulators are used. If required by users, an option is to increase slightly the charge to overcome the pulse energy.

Short temporal delays, below 1 ps, can be realized by using two bunches produced at the photo-cathode within the same RF cycle, by means of shaping the photo-cathode laser pulse, and then accelerating in the same bucket and compressing. This configuration has already been explored in simulations because of interest for electron beam driven plasma wakefield acceleration, as the first bunch can act as driver and the second as witness (Björklund Svensson *et al.*, 2017 ▸). This scheme will allow to produce pulses with a small energy difference, giving a small wavelength separation, but both pulses can reach high pulse energy. The tunability is, however, limited because the beam energy and time separation are coupled during the acceleration (Marinelli *et al.*, 2015 ▸).

## 7. Beamline design   

The scientific experiments to be performed at the SXL can be broadly divided into two, quite distinct, categories: time-resolved microscopy and photon energy-resolved spectroscopy. In the first, femtosecond or even sub-femtosecond FEL pulses are used to follow the temporal dynamics of the system in question. Meanwhile, in the second category, sub-eV energy resolution is employed. To enable both categories, two photon beamlines are designed for the SXL facility.

**Figure 11:**
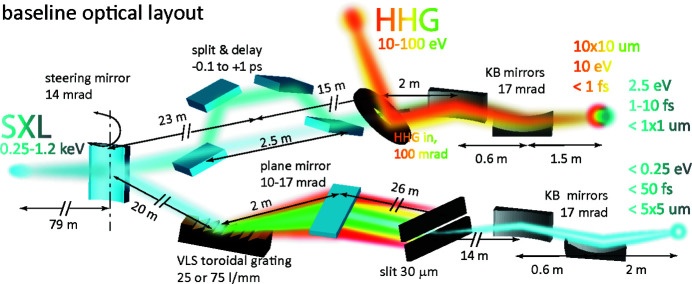
Conceptual layout of the two beamlines, one monochromatic and one pink.

An overview of the beamlines is shown in Fig. 11 ▸. At the first mirror, the FEL beam is steered into either the pink or the monochromatic beamline. The pink beamline delivers short pulses, high pulse energies and high intensities, while the monochromatic beamline provides X-ray radiation with a narrow bandwidth. The main output parameters of the beamlines at 248 eV (5 nm) and 1240 eV (1 nm) are presented in Table 4 ▸.

In the pink beamline, the FEL beam is focused onto the sample in an endstation using a set of Kirkpatrick–Baez (KB) mirrors. For single-color, pump–probe experiments, the X-ray beam can be split into two pulses using a variable split–delay system. Furthermore, laser pulses from a gas-based HHG source can be incoupled via a holey mirror for pump–probe experiments. In the monochromatic branch, the X-ray beam is dispersed by a VLS (variable line spacing) toroidal grating with simultaneous focusing in the vertical plane onto the exit slit. The focusing in the horizontal plane is accomplished with the steering mirror, M_s_. Then, the beam is refocused by means of KB mirrors onto the sample.

Table 5 ▸ presents details of the main optical components. Different choices of the curvature of the steering mirror M_s_ for the pink beam enable a flexible design. By using a flat M_s_ mirror, the pink beam can be expanded all the way to the KB mirrors, which focuses the X-rays onto the sample. The long entrance arm permits the generation of extremely small spot sizes at the sample, below 1 µm FWHM. Recall that, in the approximation of geometrical optics, the demagnification factor is given by the ratio of the length of the exit arm to the length of the entrance arm of the focusing system. For FEL operations that produce large divergence, such as the low-charge/short-pulse mode, a focusing elliptical M_s_ can be used that creates an intermediate focus between M_s_ and the KB mirrors. This allows reducing the beam size below the aperture limit of the KB mirrors and may also be useful for producing a small beam size at diagnostics positioned just before the KB mirrors, in order to increase their resolution.

The damage thresholds of the optical components have been taken into account. In order to assess the maximum pulse energy that the optics should tolerate we have estimated the maximum pulse energy produced by a stochastic FEL process over a ten year period of operation as roughly 1.5 times the average pulse energy. In addition, we assumed a 20% uncertainty of the start-to-end FEL simulations and included a safety margin for the optical design of 20%. All optical components will use silicon as bulk material thanks to high and quite uniform transmission in the entire 0.25–1.24 keV photon energy range without any absorption peaks. The damage threshold of silicon is ∼0.73 eV atom^−1^ (Koyama *et al.*, 2016 ▸) and all optical components will be exposed to a dose below this value using the estimated maximum pulse energy and safety margins described above.

## 8. Outlook and upgrades   

The SXL project will start with the basic set-up described above, *i.e.* SASE plus tapering, but developments of the system are already prepared. Phase 2 will explore more advanced concepts than SASE within three target areas: improved two-color/two-pulses tuning, longitudinal coherence/seeding and ultra-short (hundreds of attoseconds) pulses.

### 8.1. Improved two-color/two-pulses and self-seeding   

From the user community there is a strong push towards two-color/two-pulses with high tunability in terms of wavelengths and temporal delay. To tune the delay between the two pulses through a zero crossing, the split undulator scheme can switch wavelength between the two undulator parts, but this will influence the properties and source points of the two pulses. To reach negative delay times in a smooth tuning, optical techniques are necessary. One method is to separate the two optical pulses transversely, and put an optical delay in the beamline. The separation can be achieved by running the electron beam through the second part of the undulator chain with an offset in angle and/or position. While the distance to the source point will be different, the direction to the source point can be made to overlap. A second option for tuning through zero-delay is to complement the electron chicane, installed in Phase 1 after the tenth undulator, with an optical system to be used both to delay the optical pulse and for soft X-ray self-seeding (SXSS) (Ratner *et al.*, 2015 ▸). While the systems technically are similar, the optics is different as SXSS requires one of the optical elements to be a grating. We believe that this can be accomplished in the same system by exchangeable optics. By using a fixed electron pulse delay, say 100 fs, the two optical pulses can more freely be chosen to overlap or arrive either before or after.

### 8.2. Echo-enabled harmonic generation   

Seeding with an external laser offers several advantages over SASE such as improving the longitudinal coherence, fixing the central wavelength and synchronization of the FEL pulse with an external laser at the experiments. Among the laser seeding techniques, EEHG (Xiang & Stupakov, 2009 ▸) is to date the only method we found that when applied to the SXL electron beam is capable of reaching, at least partly, the wavelength range of the SXL. Initial estimates including EEHG seeding (Pop & Werin, 2019 ▸) showed promising results. However, in standard mode the SXL is providing electron pulses with an energy chirp on the order of 0.5 MeV fs^−1^ after the last bunch compressor (BC2). Ongoing studies indicate that this is still within tolerance for the EEHG scheme, but further simulations regarding the effect of CSR and chromatic effects are needed. Indeed EEHG is at the moment considered only for the wavelength range 3 to 5 nm, and simulations results at 5 nm show a bandwidth reduction of one order of magnitude with respect to SASE.

### 8.3. HB-SASE and chirp removal   

Another feasible way of improving the coherence, in the SXL wavelength range, is through high-brightness SASE (HB-SASE) (McNeil *et al.*, 2013 ▸). In this method the optical mode is coupled over a longer region of the electron pulse than standard SASE, and the longitudinal coherence length is thus increased. In practice this is done by utilizing shorter undulators and delay-chicanes between every undulator. The SXL will be based on 2 m undulators and the phase shifting chicanes, necessary for ordinary FEL operation, will be strong enough to also act as delay chicanes in HB-SASE mode. While simulations show that this mode is possible in standard operation, to reach a strong improvement in bandwidth narrowing and coherence improvement, the energy chirp in the electron beam has to be further decreased. As described above, methods to remove the chirp are being studied, initially based on overcompression.

### 8.4. Attosecond pulses   

Ultra-short pulses (hundreds of attoseconds) are also highly interesting for certain experiments and will be a feature of the SXL project, especially in combination with the pump–probe capabilities. The obvious path for the future of the SXL is to further explore the capabilities of the accelerator system to create extremely short pulses. In double-achromat compressors, the presence of a central peak and the absence of horns in the tails can be used to achieve bunches below 1 fs. How such an operation mode can be implemented and controlled is still under development. Critical factors are to match the diagnostics and how to prevent bunch elongation during the FEL process. Nevertheless, double-achromat compressors are less sensitive to klystron modulator high voltage jitter (which is particularly relevant in view of the short bunches).

In parallel the SXL undulator layout is intended to be flexible enough to take advantage of concepts under development in short pulse generation, such as the generation of attosecond pulse trains (Maroju *et al.*, 2020 ▸).

## 9. Conclusions   

In this paper we presented the design of the FEL in the SXL project at the MAX IV laboratory with emphasis on the Phase 1 of the project. The FEL design targets operation in the initial phase in SASE mode complemented with tapering of the undulators. This will satisfy a broad range of the science case.

The double-achromat bunch compressors can naturally linearize the pulse and provide strong compression creating short bunches for the FEL process in SXL. Two-pulse/two-color operation will be provided by an electron delay chicane in the middle of the undulator chain. This chicane can later be complemented with an optical system to allow for both tuning pulse separation ‘through zero time’ and to provide seeding through self-seeding. Longer time delays will be available through acceleration of two electron pulses in adjacent RF buckets. The undulator length and the layout of the intrasections (the presence of strong magnetic chicanes) will allow flexibility in the future to explore more advanced concepts like HB-SASE, seeding using EEHG and generation of attosecond pulse trains.

## Figures and Tables

**Table 1:** Electron beam parameters for long (1A) and short (1B) pulses at the undulator entrance

Pulse	1A	1 B
Bunch charge (pC)	100	10
Peak current (A)	4500	3900
Bunch FWHM length (fs)	11	1.3
Energy chirp (MeV fs^−1^)	0.4	5
Slice RMS norm. emittance (*x*, *y*) (mm mrad)	0.37, 0.38	0.19, 0.14
Slice RMS energy spread (MeV)	0.3	0.8

**Table 2:** Undulators main parameters

Undulator length	2 m
Period length	40 mm
Wavelength range	5–1 nm
Effective *K*_*x*,*y*_ range (linear mode)	3.9–1.2
Polarization	Linear/helical

**Table 3:** Simulated FEL performance for the long-pulse/high-charge case (1A)

	1 nm with taper	5 nm with taper
Active length (m)	40	30
Pulse energy (mJ)	0.66	1.5
Peak power (GW)	50	56
Photons per pulse	3.3 × 10^12^	3.8 × 10^13^
Pulse duration (fs) (FWHM)	14	26
Bandwidth (%) (FWHM)	0.8	1.2
Source radius *x*/*y* (µm)	57/54	149/145
Source divergence *x*/*y* (µrad)	8.5/8.1	32/32
Source location *x*/*y* (m)	−10/−9.9	−8.7/−8.8

**Table 4:** Parameters of the final FEL beam, after passing the beamlines, using tapered FEL operation The distributions are given in terms of FWHM. Note that the parameters corresponding to the ‘Mono, short pulses’ and ‘Mono, high resolution’ columns are achieved with different gratings (25 lines mm^−1^ and 75 lines mm^−1^, respectively). The former will typically be used for longer wavelengths, and the latter for shorter, in order to provide a high transmission over the full spectral range.

Parameter	Pink		Mono, short pulses		Mono, high resolution	
Grating	–	–	25 lines mm^−1^, 0.15° blaze		75 lines mm^−1^, 0.25° blaze	
Wavelength (nm)	1	5	1	5	1	5
Transmission (%)	71	61	0.023	0.54	0.58	0.05
Pulse energy (µJ)	469	915	0.2	8.1	3.8	0.8
Photons pulse^−1^ × 10^10^	235	2300	0.08	20	1.9	1.9
Beam size (µm)	0.75 × 1.1	1.9 × 2.7	2.5 × 5	4.9 × 6.2	2.5 × 5	4.9 × 5.8
Pulse stretching (fs)	–	–	6	55	16	175
Resolution (meV)	6200	1740	650	71	260	23

**Table 5:** The main optical components

Parameter	Mode	M_s_	G_1_/G_2_	M_m_	KB_1_	KB_2_
Shape	Pink	Plane/elliptical	–	–	Plane-elliptical	Plane-elliptical
	Mono	Toroidal	Spherical	Plane	Plane-elliptical	Plane-elliptical

## Footnotes

1 We define as light ‘effective emittance’ the product of beam size and divergence.
